# 390. Lenacapavir Adherence and Effectiveness Over 2 Years of Use in the OPERA Cohort

**DOI:** 10.1093/ofid/ofaf695.128

**Published:** 2026-01-11

**Authors:** Karam Mounzer, Laurence Brunet, Ricky K Hsu, Philip C Lackey, Michael G Sension, Michael B Wohlfeiler, Keith Dunn, Jennifer S Fusco, Gregory P Fusco

**Affiliations:** Philadelphia Fight Community Health Centers, Philadelphia, Pennsylvania; Epividian, Inc., Durham, North Carolina; AIDS Healthcare Foundation/ NYU School of Medicine, New York, NY; N/A, Winston-Salem, North Carolina; can community health, Miami Beach, FL; AIDS Healthcare Foundation, Miami, Florida; Gilead Sciences, Foster City, California; Epividian, Inc., Durham, North Carolina; Epividian, Inc., Durham, North Carolina

## Abstract

**Background:**

The first in class capsid inhibitor lenacapavir (LEN) is a twice yearly set of two injections used with an optimized background regimen for heavily treatment-experienced individuals with multidrug resistant HIV-1 infection. We describe real-world LEN injection adherence and effectiveness in a US cohort.

**Figure:**
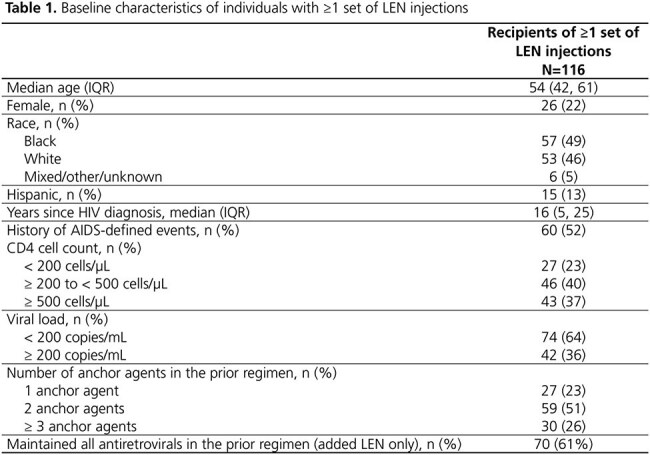

**Figure:**
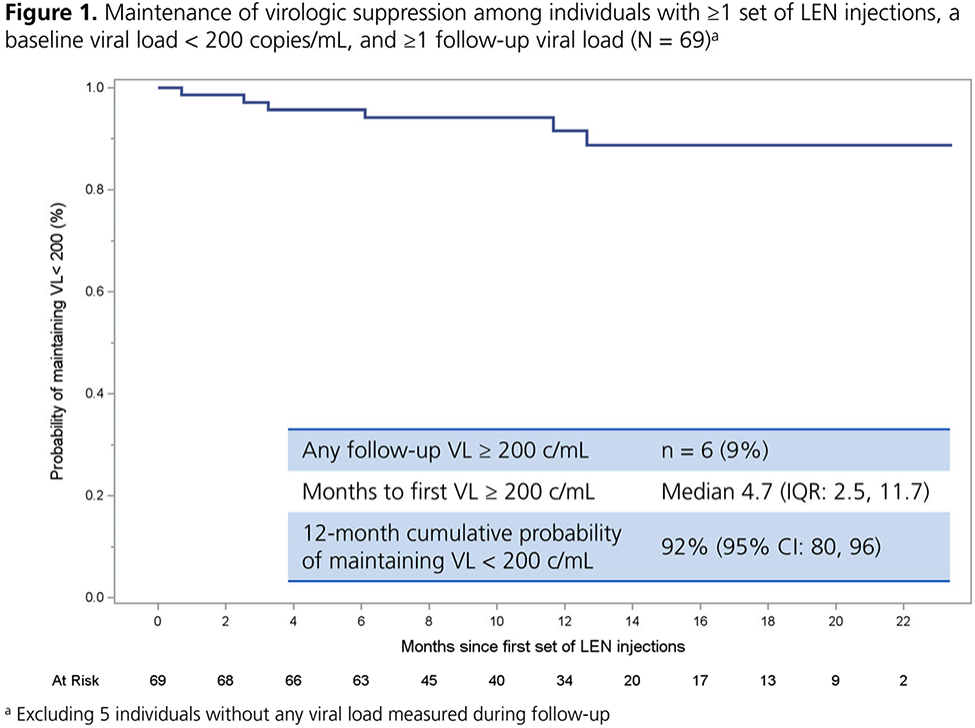

**Methods:**

From electronic health records in the OPERA cohort, all ART-experienced adults with HIV starting a LEN-containing regimen between 22DEC2022 and 30SEP2024 were followed through 31DEC2024. Maintenance or achievement of virologic suppression (viral load < 200 copies/mL) over time on LEN was assessed with Kaplan Meier methods among those with viral load(s) measured during follow-up. Adherence to injection windows among individuals with ≥ 2 sets of injections was defined based on the time elapsed since the prior set of injections (early: < 24 weeks, on-time: ≥ 24 to ≤ 28 weeks, late: > 28 weeks). Discontinuation was defined as the absence of injections between the end of the 28 weeks window and study end.

**Figure:**
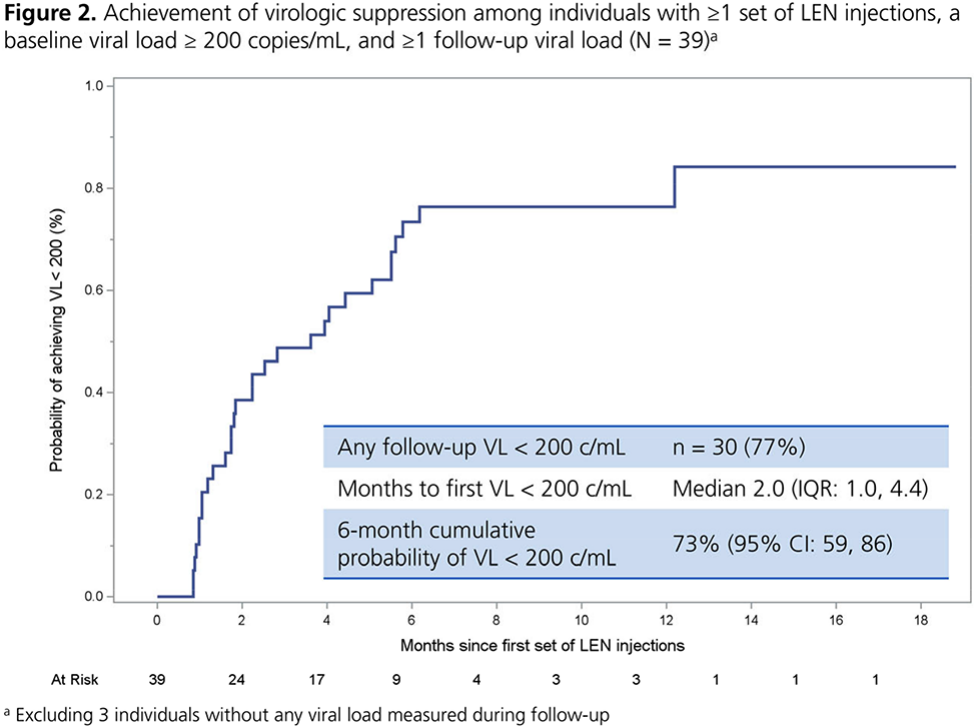

**Figure:**
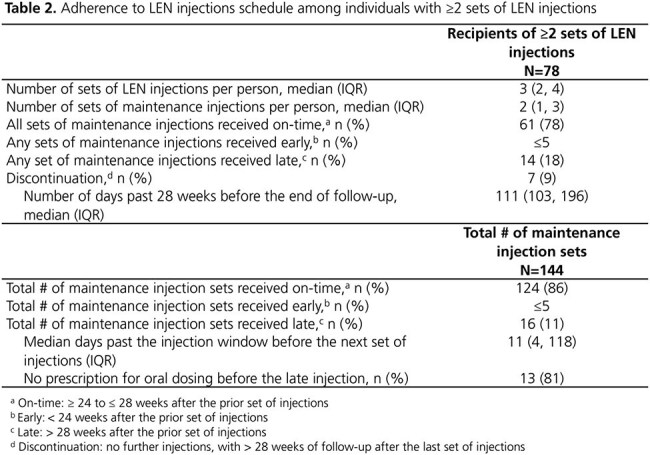

**Results:**

A total of 116 individuals received ≥ 1 set of LEN injections (median 3 sets over follow-up; IQR: 2, 4). At the first set, 22% were women, and most had moderate to high CD4 cell counts and low viral loads (Table 1). Of the 61% who added LEN to their prior regimen, 51% had no further changes after LEN initiation. The cumulative probability of maintaining suppression throughout follow-up was high (92%; 95% CI: 80, 96) among 69 individuals starting LEN with a viral load < 200 copies/mL (Figure 1). The cumulative probability of achieving suppression was also high (73%; 95% CI: 59, 86) among 39 individuals starting LEN with a viral load ≥ 200 copies/mL (Figure 2). Among 78 individuals who received ≥ 2 sets of LEN injections, 78% received all sets on time and 18% received ≥ 1 set a median of 11 days past the injection window; 9% discontinued (Table 2).

**Conclusion:**

In this cohort representative of routine clinical care in the US, adherence to the injection schedule was good, most delays observed were short, and the majority of users remained on LEN through study end. Most LEN users experienced favorable virologic outcomes regardless of baseline viral load. LEN may be an option for treatment experienced individuals who could benefit from a long-acting injectable agent from a novel antiretroviral class.

**Disclosures:**

All Authors: No reported disclosures

